# Maximizing *N*-Nitrosamine Rejection via RO Membrane Plugging with Hexylamine and Hexamethylenediamine

**DOI:** 10.3390/nano14131117

**Published:** 2024-06-28

**Authors:** Silvia Morović, Katarina Marija Drmić, Sandra Babić, Krešimir Košutić

**Affiliations:** 1 Department of Physical Chemistry, Faculty of Chemical Engineering and Technology, University of Zagreb, Trg Marka Marulića 20, 10000 Zagreb, Croatia; smorovic@fkit.unizg.hr; 2 Department of Analytical Chemistry, Faculty of Chemical Engineering and Technology, University of Zagreb, Trg Marka Marulića 20, 10000 Zagreb, Croatia; kdrmic@fkit.unizg.hr (K.M.D.); sbabic@fkit.unizg.hr (S.B.)

**Keywords:** *N*-nitrosamines, RO membranes, molecular plugging, membrane modification, water reuse

## Abstract

The rapid expansion of urban areas and the increasing demand for water resources necessitate substantial investments in technologies that enable the reuse of municipal wastewater for various purposes. Nonetheless, numerous challenges remain, particularly regarding disinfection by-products (DBPs), especially carcinogenic compounds such as *N*-nitrosamines (NTRs). To tackle the ongoing issues associated with reverse osmosis (RO) membranes, this study investigated the rejection of NTRs across a range of commercially available RO membranes. In addition, the research aimed to improve rejection rates by integrating molecular plugs into the nanopores of the polyamide (PA) layer. Hexylamine (HEX) and hexamethylenediamine (HDMA), both linear chain amines, have proven to be effective as molecular plugs for enhancing the removal of NTRs. Given the environmental and human health concerns associated with linear amines, the study also aimed to assess the feasibility of diamine molecules as potential alternatives. The application of molecular plugs led to changes in pore size distribution (PSD) and effective pore number, resulting in a decrease in membrane permeability (from 5 to 33%), while maintaining levels suitable for RO processes. HEX and HDMA exhibited a positive effect on NTR rejection with ACM1, ACM5 and BW30LE membranes. In particular, NDMA rejection, the smallest molecule of the tested NTRs, with ACM1 was improved by 65.5% and 70.6% after treatment with HEX and HDMA, respectively.

## 1. Introduction

While around 50% of wastewater from human activities is discharged untreated into rivers or oceans, with significant environmental and health impacts [1], almost two thirds of the world’s population suffer from severe water shortages for at least one month a year [2]. The wastewater reuse practices for agricultural and industrial purposes are therefore becoming increasingly important and play a significant role in mitigating water shortages [3]. In 2021, the European Environment Agency (EEA) reported that over 86.14% of wastewater treatment plants across Europe use tertiary treatment methods that include both physical and chemical processes to eradicate harmful microorganisms from wastewater [4]. Typically, this process involves filtration followed by a disinfection step. While secondary and tertiary treatment are generally considered sufficient for environmental protection, they have shown inadequacies when dealing with emerging contaminants (CECs) [5,6]. CECs enter surface waters via municipal wastewater treatment plants, raising concerns due to their proven detrimental effects on the environment and human health [7]. In addition, existing studies suggest that CECs found in water may serve as precursors for the generation of disinfection by-products (DBPs) when the water is disinfected using chlorine and chloramines [8,9]. Of particular concern among these compounds is the presence of *N*-nitrosamines (NTRs), which are formed by oxidation reactions with secondary or tertiary amines in the presence of a nitrosating agent and are classified as “possibly carcinogenic to humans” [10]. The removal of NTRs from water has proven to be extremely difficult in practical applications. Notwithstanding this, compliance with stringent standards for the use of reclaimed water or, in the context of drinking water, prior to release into distribution systems, is essential due to the carcinogenic nature of NTRs even at very low concentrations [11]. Despite their reputation as cutting-edge water treatment processes capable of meeting stringent quality criteria, reverse osmosis (RO) and nanofiltration (NF) technologies face a significant limitation in effectively separating uncharged small molecules like *N*-nitrosodimethylamine (NDMA). For instance, Fujioka et al. conducted thorough investigations into the retention of NTRs by RO membranes. In one of their studies, they compiled all available laboratory-scale data and found that the retention of NDMA by various RO membranes was no more than 70% [12,13]. Considering the absence of viable alternatives to thin film composite polyamide (TFC-PA) membranes, the concept of adapting their selectivity to effectively target neutral molecules while retaining other advantageous properties is of great interest [14]. Extensive research on improving the selectivity of RO membranes for small neutral molecules can be found in the literature. One strategy entails the addition of an additional layer onto the PA membrane in order to maximize its selectivity [15,16,17,18]. Another approach is to integrate functionalized nano materials such as metal–organic frameworks [19], silica [20] and carbon nanotubes [21] into polyamide membranes, aiming to modify their properties. A third approach focuses on fine-tuning the rejection properties of the PA selective layer by incorporating or binding molecules known as molecular plugs [14]. This approach carries the potential to narrow pores within the PA layer and thus induce the size exclusion mechanism, as well as the possibility of causing changes in chemical composition or polarity. Molecular plugging is a technique that aims to tighten nanopores of RO membranes by incorporating small hydrophobic molecules, thus effectively reducing the permeation of the targeted solutes [22]. This method is promising, especially for applications where the inherent selectivity of PA membranes was insufficient. For instance, Shultz et al. have shown that the use of aliphatic amines with carbon chains of 10 to 12 atoms, whether by covalent bonding or simple adsorption, can reduce boron permeability by a factor of 2 to 4 while retaining the membrane’s ability to retain salts. However, this improved selectivity was accompanied by a reduction in water permeability [23]. In another study by Li et al., aliphatic amines were used in two ways: first, as hydrophobic barriers by binding to residual chloride groups on the membrane surface, and second, as molecular plugs incorporated within the PA networks. This dual approach led to a remarkable improvement in boron rejection, which increased from 80.7% to 90.5%. This improvement is attributed to the increased steric hindrance caused by the immobilized amine plugs and the synergistic adjustment of the hydrophobic interactions [24]. Considering that NDMA and boric acid have similar rejection mechanisms, it is anticipated that the use of molecular plugging as a modification method for RO membranes will lead to positive outcomes in the rejection of NDMA. Fujioka et al. investigated the effectiveness of plugging RO membranes with linear amines, amides and epoxides to improve NDMA and salt rejection. In general, the use of amines or epoxides for plugging resulted in reduced water permeability and increased conductivity rejection. The rejection of NDMA showed a direct correlation with the size of the amines. Particularly noteworthy was the achievement of an 80% NDMA rejection rate when the membrane was plugged with dodecylamine during the treatment of effluent from a membrane bioreactor, compared to only 42% for pristine membrane [22].

To address the ongoing challenges encountered by RO membranes, this research aimed to investigate the NTRs rejection of various commercially available RO membranes and to improve the rejection rates by integrating molecular plugs into the nanopores of the PA layer. Linear-chain amines have been identified as effective molecular plugs that can efficiently penetrate and incorporate into the nanopores, thereby enhancing the plugging effect thus improving the rejection of NDMA, the smallest NTRs molecule. This study focused on hexylamine (HEX) and hexamethylenediamine (HDMA) to investigate whether the incorporation of additional positively charged ${–NH}_{3}^{+}$ groups could improve the integration of the molecular plugs, i.e., amines, into the nanopores of the membranes and thus improve the rejection of NDMA. Considering the environmental and human health risks associated with linear amines, we also sought to evaluate the potential of diamine molecules as alternative substitutes.

## 2. Materials and Methods

### 2.1. Chemicals and Materials

The chemicals used were of analytical grade: hexamethylenediamine (98+%, Thermo Fisher Scientific, Kandel, Germany), hexylamine (>99%, TCL, Zwijndrecht, Belgium), sodium chloride (p.a., Lach-Ner, Neratovice, Czech Republic). *N*-nitrosamines used were as follows: *N*-nitrosodimethylamine (5000 μg/mL in methanol, LGC Standards, Kulmbach Germany), *N*-nitrosodiethylamine (99%, Aldrich, St. Louis, MO, USA), *N*-nitrosomorpholine (Supelco, Darmstadt, Germany), *N*-nitrosopiperidine (5000 μg/mL in methanol, Supelco, Darmstadt, Germany) and their physicochemical properties are listed in Table 1. HPLC-grade acetonitrile (J. T. Baker, Deventer, The Netherlands), methanol (J. T. Baker, Deventer, The Netherlands), dichloromethane (T.T.T., Sveta Nedjelja, Croatia) and ultra-pure water prepared using the Milli-Q^®^ Reagent Grade Water System (Millipore Corporation, Bedford, MA, USA) were used for SPE and chromatographic analysis. The organic compounds chosen as markers included trimethylene oxide (97%, Acros Organics, Morris Plains, NJ, USA), 1,3-dioxolane (99.8%, Sigma-Aldrich, St. Louis, MO, USA), 1,4-dioxane (99.8%, Sigma-Aldrich, St. Louis, MO, USA), 12-crown-4 (98%, Fluka, Buchs, Switzerland), 15-crown-5 (98%, Merck-Schuchardt, Hohenbrunn, Germany), and 18-crown-6 (99.5%, Fluka, Buchs, Switzerland). Coconut Charcoal SPE cartidges (2 g/6 mL tube) were purchased from Merck (Darmstadt, Germany). Additionally, commercial RO membranes BW30LE and XLE (DOW FILMTEC™, Wilmington, DE, USA), UTC-73AC (Toray™, Poway, CA, USA), ACM1, and ACM5 (TriSep™, Goleta, CA, USA) were provided by Sterlitech Corporation (Auburn, WA, USA) in the form of flat sheets and were kept dry prior to usage. The manufacturer-provided nominal characteristics of these membranes are detailed in Table 2.

### 2.2. Membrane Plugging Procedure

The RO membranes underwent plugging by being exposed to the plugging solution using the specified laboratory-scale RO system. This step included the use of a 2 mM solution of the molecular plug, either hexylamine or hexamethylenediamine (the physicochemical characteristics of the chosen amines are provided in Table 3.

### 2.3. Characterization of TFC-PA Membranes

#### 2.3.1. RO Performance

**Figure 1:**
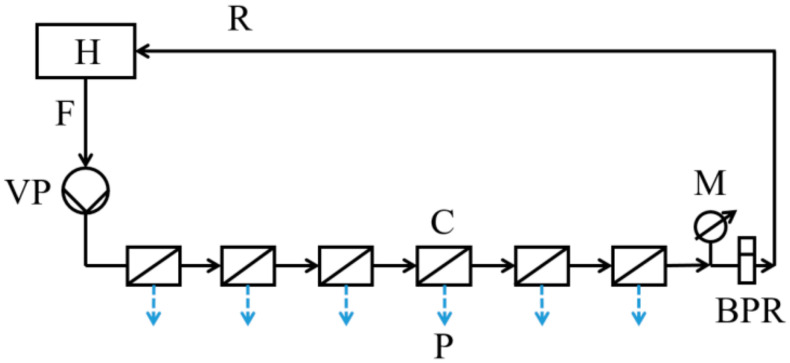
Schematic representation of the RO set up. H: hold-up tank, VP: high pressure pump, M: manometer, BPR: back pressure regulator, C: membrane cells. F, P and R stand for feed, permeate and retentate streams, respectively. The blue arrows represent the permeate stream.

RO experiments were conducted utilizing a laboratory-scale cross-flow filtration setup comprising six stainless steel membrane units connected in series (Figure 1).

Each membrane unit comprises two components: the upper part, which remains stationary and serves as a high-pressure chamber housing the inlet and outlet for the pressurized fluid, and the detachable lower part containing a porous stainless steel plate. Membrane coupons, with an area of 13 cm^2^, were cut from flat membrane sheets and positioned on top of the porous stainless steel plate within the lower section of the membrane units.

Before experiments, the membranes underwent compaction at a pressure ranging from 20% to 40% higher than the operating pressure for a duration of 2 h. Subsequently, the working pressure was adjusted to 15 bar. All experiments were conducted at room temperature, specifically 22.0 ± 2.0 °C. The feed solution circulated through the cells at a flow rate of 0.7 L min^−1^. Water permeability was determined by collecting and weighing the permeate. Water permeability was adjusted to 25.0 °C using the relative viscosity and density of pure water. The performance of the modified RO membranes was assessed using a solution comprising NaCl at 500 mg L^−1^, along with NDMA, NDEA, NPIP, and NMOR, each at a concentration of 100 μg L^−1^ in a buffer solution prepared from potassium dihydrogen phosphate (Lach-Ner, Neratovice, Czech Republic, MW = 136.09 g mol^−1^) and dipotassium hydrogen phosphate (VWR Chemicals BDH Prolabo, Rosny-sous-Bois, France, MW = 174.18 g mol^−1^) to maintain a pH of 7.2. The concentration of each NTRs was determined via HPLC-DAD. Salt rejection was evaluated by measuring the conductivity of both the permeate and feed solutions using a Lab 960 instrument from SCHOTT Instruments, Mainz, Germany. In order to determine the rejection factor for each marker employed in estimating the pore size distribution (PSD), solutions were initially prepared with a concentration of 100 mg L^−1^. The concentrations of markers were measured using a Total Organic Carbon Analyzer (TOC-VWS, Shimadzu, Kyoto, Japan). The rejection factors (*R*) were computed using Equation (1), where *c*_p_ and *c*_f_ denote the concentrations of solutes in the permeate and feed solutions, respectively.

*R*/% = (1 − *c*_p_/*c*_f_)·100 (1)

#### 2.3.2. ATR-IR Spectral Analysis

FTIR analysis was utilized to ascertain the chemical compositions of RO membranes. Spectra were obtained at room temperature through a Perkin-Elmer Spectrum One instrument, featuring an ATR module, covering the frequency range of 650–4000 cm^−1^ with 4 scans and a resolution of 4 cm^−1^. Before analysis, membrane samples underwent a 24-h drying process. Subsequently, the FTIR spectra were analyzed using OriginPro 8.5.0 SR1 software.

#### 2.3.3. Contact Angle

Contact angle assessments were conducted using the OCAH 200 Data Physics contact angle system. Employing the sessile drop method, 5 μL of water droplets was deposited onto the surfaces of different RO membranes at room temperature. To mitigate potential factors like temperature and humidity impacting contact angle readings, measurements were synchronized and carried out simultaneously. The reported contact angle values for each membrane represent the average of 10 measurements.

#### 2.3.4. Scanning Electron Microscopy

Before analysis, dry membrane samples underwent a 90-s argon plasma coating with an Au/Pd alloy to enhance their electrical conductivity. Microscopic imaging was conducted using a TESCAN Vega3 SEM Easyprobe electron microscope operating at 10 kV voltage.

#### 2.3.5. Solid Phase Extraction Procedure (SPE)

A SPE vacuum manifold (24-port model) was used (Supelco, Gillingham, Dorset, UK). SPE cartridges were conditioned using dichloromethane, followed by methanol (10 mL) and deionized water. A 100 mL volume of sample was then applied to the cartridge. Then, the cartridges were dried for 10 min with a vacuum. Following that, the retained compounds were eluted using 2 × 5 mL of dichloromethane. The final volume of the extract was 10 mL. Solvent evaporation from extracts after solid phase extraction was carried out on a Rotavapor R-114 Büchi (Flawil, Switzerland) for 5 min at 25 °C.

#### 2.3.6. High-Performance Liquid Chromatography Analysis

Quantitative determination of NTRs (NDMA, NDEA, NMOR, NPIP) in feed solutions and permeate was performed using an Agilent Series high-performance liquid chromatography (HPLC) system coupled with a diode array (DAD) detector (Agilent 1100, Santa Clara, CA, USA). The HPLC system consists of a mobile phase container, a vacuum degasser, an automatic sampler, a thermostated chromatographic column compartment, and a binary pump for mobile phase delivery. Kinetex C18 chromatographic column (Phenomenex, 150 mm × 4.6 mm, 5 mm, 100 Å) was used for chromatographic separation. The mobile phase consisted of MiliQ water as eluent A and acetonitrile as eluent B in gradient elution mode. The flow rate of 0.5 mL min^−1^ and an injection volume of 20 μL were maintained throughout the analysis. The gradient elution started with 95% of eluent A and was on an isocratic hold for 7.5 min. After that, the proportion of eluent A linearly decreased to 40% over 7.5 min. The proportion of eluent A of 40% was kept on the isocratic hold for the next 10 min. The proportion of eluent A was returned in the next 5 min to the initial value (95%) and remained the same for 10 min. Targeted NTRs were detected at the wavelength of 254 nm. Instrument control, data acquisition, and evaluation were performed with ChemStation Rev. B.04.02 SP1 software (Agilent, Santa Clara, CA, USA).

#### 2.3.7. Pore Size Distribution

For determining the pore size distribution, we employed the Surface Force–Pore Flow (SF-PF) theoretical model, initially introduced by Sourirajan and Matsuura and detailed elsewhere [25,26,27]. This model posits cylindrical pores within the PA layer and characterizes solute–membrane interactions with water using Lennard-Jones surface potential functions, representing both intermolecular repulsion and dispersion. The calculation method aims to identify a pore size distribution that minimizes the difference between the observed and predicted rejections and permeation velocities of specific disc-shaped molecules.

## 3. Results

### 3.1. Permeability

**Figure 2:**
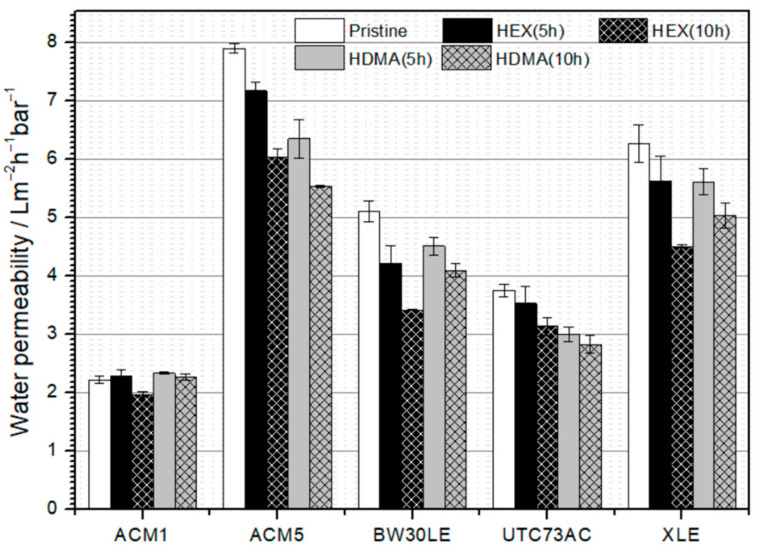
Pure water permeability of selected pristine and modified RO membranes. The error bars indicate the average values and the range of duplicated results.

The water permeability data for selected RO membranes is depicted in Figure 2. It is evident that the pristine RO membrane ACM1 exhibits the lowest water permeability, whereas the ACM5 membrane demonstrates nearly four times higher water permeability, likely due to differences in the structure of the PA layer. Following treatment of membranes with HEX plugging solution for 5 and 10 h, ACM5, BW30LE, and XLE membranes exhibit a similar trend. The water permeability decreased by 9.2%, 17.7%, and 10.2%, respectively, after 5 h of plugging, and by 23.5%, 33.2%, and 28.1%, respectively, after 10 h of plugging. The water permeability of ACM1 was increased by 2.9% after 5 h of plugging and reduced by 11.1% after 10 h with HEX solution. For the UTC73AC membrane, a decrease in water permeability of 5.7% and 16.3% was observed after 5 and 10 h of plugging with HEX solution, respectively. Moreover, after treating the membranes with HDMA plugging solution for 5 and 10 h, water permeability decreases by 19.6% and 20.0%, respectively, after 5 h of plugging, and by 29.9% and 24.5% after 10 h of plugging in the case of the ACM5 and UTC73AC membranes. For the BW30LE membrane, following 5 h of plugging with HDMA solution, water permeability dropped by 11.7%, whereas after 10 h of plugging, it decreased by 19.8%. It is noteworthy that the water permeability reduction is considerably less compared to plugging with HEX solution. On the other hand, it was shown that there was a slight increase in water permeability after 5 h of plugging with HDMA solution for ACM1 with an increase of 5.1%. The trend remains consistent for the ACM1 membrane after 10 h of plugging, showing a 2.3% increase in water permeability. The water permeability of the XLE membrane was 19.7% lower than that of the pristine membrane after 5 h and 24.5% lower after 10 h of plugging with HDMA.

### 3.2. Rejection of NTRs and NaCl Rejection

**Figure 3:**
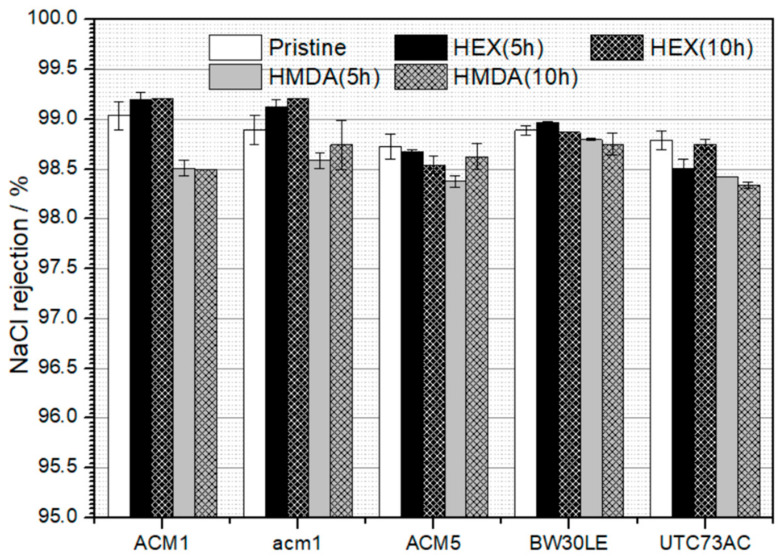
NaCl rejection rates for specific RO membranes. The error bars show the mean values and the variation range of the duplicated results.

The NaCl rejection remained stable and did not exhibit notable alterations compared to the pristine membranes, with variations ranging from 0.02% to 1.2% (Figure 3). The highest variation observed in conductivity rejection was 1.2% for the XLE membranes.

**Figure 4:**
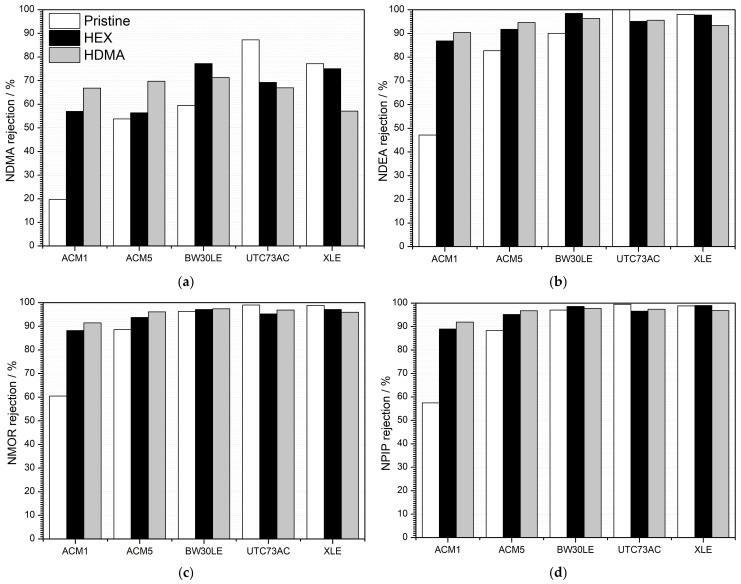
Rejection rates of (**a**) NDMA, (**b**) NDEA, (**c**) NMOR and (**d**) NPIP for pristine and modified RO membranes.

Utilizing HEX as a plugging solution demonstrated a positive effect on certain membranes, enhancing the efficiency of NTRs removal. As shown in Figure 4, the rejection factors for ACM1, ACM5 and BW30LE membranes increased significantly for NDMA (65.5%, 4.6% and 22.9%, respectively), NDEA (45.7%, 9.9% and 8.6%, respectively), NMOR (31.5%, 5.5% and 0.8%, respectively) and NPIP (35.4%, 7.2% and 1.5%, respectively) after 10 h of HEX treatment compared to the corresponding pristine membranes. Conversely, a clearly negative effect of HEX as a plugging solution can be observed in the UTC73AC and XLE membranes. Exposure of these membranes to the HEX solution resulted in a reduction in the rejection of NTRs, with decreases observed for NDMA (25.9% and 2.9%, respectively), NDEA (5.1% and 0.3%, respectively), NMOR (4% and 1.7%, respectively) and NPIP (3.1% and 0.2%, respectively). When HDMA was used as a plugging solution (Figure 4), a positive impact on the rejection factor of NTRs was again shown, particularly for ACM1, ACM5 and BW30LE membranes. Specifically, significant improvements were observed for NDMA (70.6%, 22.8% and 16.6%, respectively), NDEA (47.9%, 12.6% and 6.5%, respectively), NMOR (33.9%, 7.8% and 1.1%, respectively) and NPIP (37.5%, 8.7% and 0.7%, respectively). However, a negative effect of HDMA on the UTC73AC and XLE membranes was observed, similar to the HEX solution. This negative impact resulted in a decrease in rejection factor for NDMA by 30.3% and 35.2%, for NDEA by 4.6% and 5.1%, for NMOR by 2.2% and 3%, and for NPIP by 2.2% and 2.1%, respectively.

### 3.3. Contact Angle

**Figure 5:**
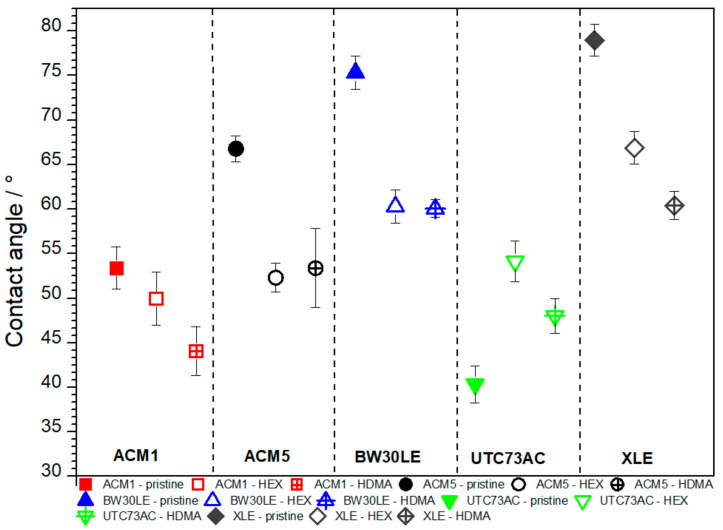
Water contact angle for pristine and modified RO membranes. The error bars show the mean values and the variation range of at least 10 measurements.

Contact angle measurements serve as a valuable tool for assessing an array of surface properties including hydrophobicity, surface wettability, surface charge, interaction energy, and other pertinent characteristics. In this study, the alterations in surface properties were also investigated by contact angle measurements with deionized water on the specified RO membranes subsequent to their exposure to plugging solutions. Notably, it is discernible that, with the exception of UTC73AC, the contact angle decreased in all cases after exposure to HEX and HDMA solutions (Figure 5). More specifically, the contact angles of ACM1, ACM5, BW30LE and XLE membranes decreased by 6.5%, 21.7%, 19.9% and 15.3%, respectively, after exposure to HEX solution. Similarly, the contact angles decreased by 17.4%, 20.1%, 20.3% and 9.7% for the same membranes after exposure to HDMA. In contrast to the trend observed for the other membranes, the UTC73AC membrane showed an increase in contact angle of 25.5% following exposure to HEX solution and 16.1% after exposure to HDMA.

### 3.4. ATR-IR

**Figure 6:**
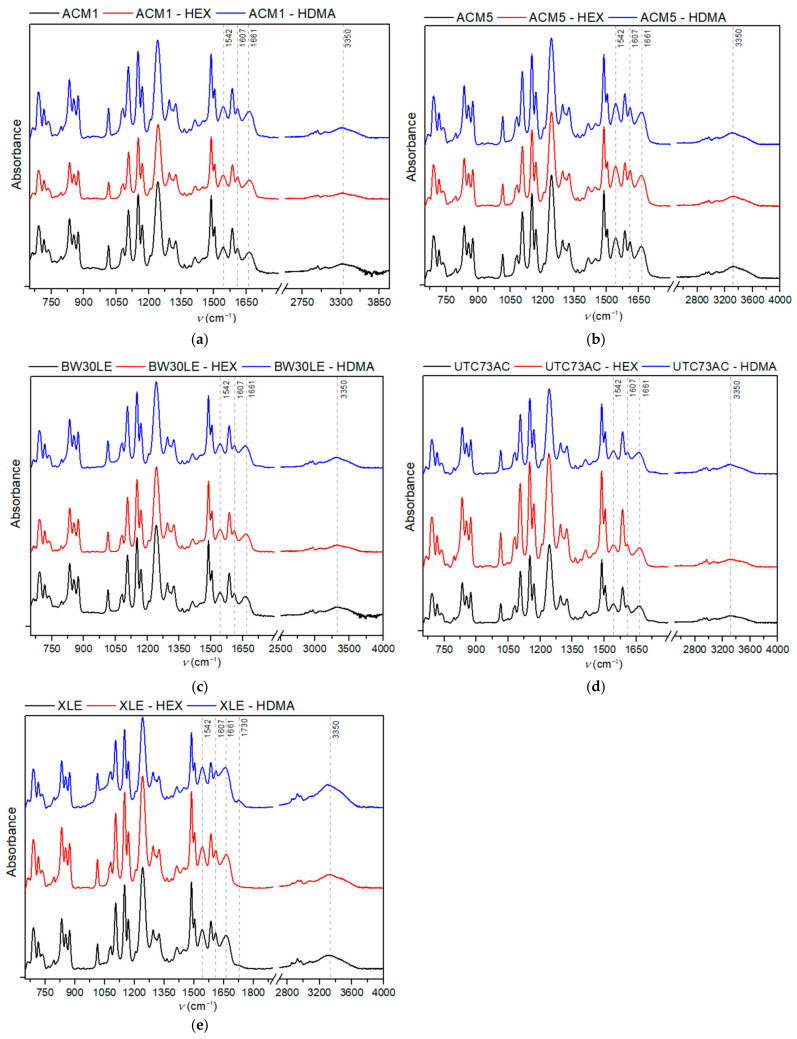
Infrared spectra of pristine and modified RO membranes; (**a**) ACM1, (**b**) ACM5, (**c**) BW30LE, (**d**) UTC73AC and (**e**) XLE.

ATR-IR was used to evaluate the chemical composition of selected pristine RO membranes and those exposed to plugging solutions. Regarding the identifiable polyamide bands, the distinctive band observed at 1661 cm^−1^ is assigned to C=O stretching, which contributes primarily to the amide I band, along with C–N stretching and the C–C–N deformation vibration in a secondary amide group. Similarly, the band at 1607 cm^−1^ is associated with either the N–H deformation vibration or the C=C stretching vibration in an aromatic amide. Furthermore, the in-plane N–H bending vibration and the N–C stretching vibration of a –CO–NH group are located at 1542 cm^−1^ [28]. As shown in Figure 6, all of aforementioned bands can be seen in the infrared spectra of pristine membranes as well as in those of the membranes that were exposed to the plugging solutions.

### 3.5. SEM

**Figure 7:**
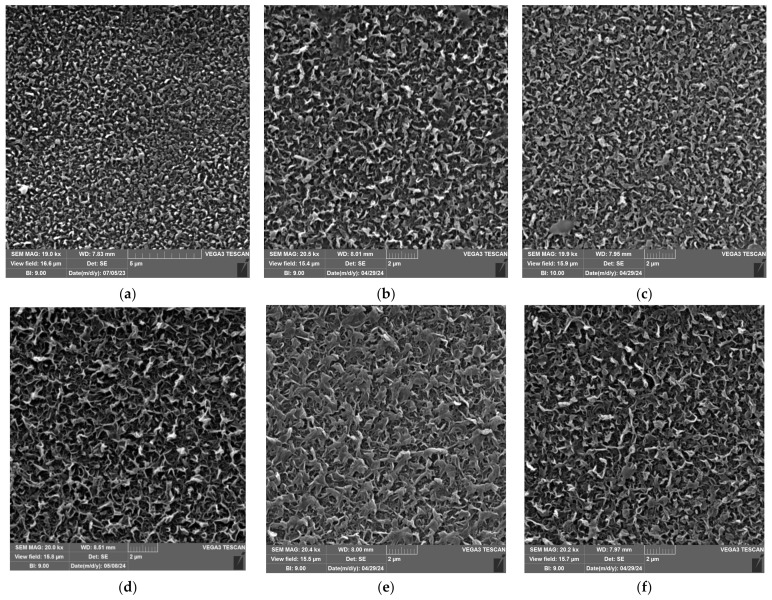

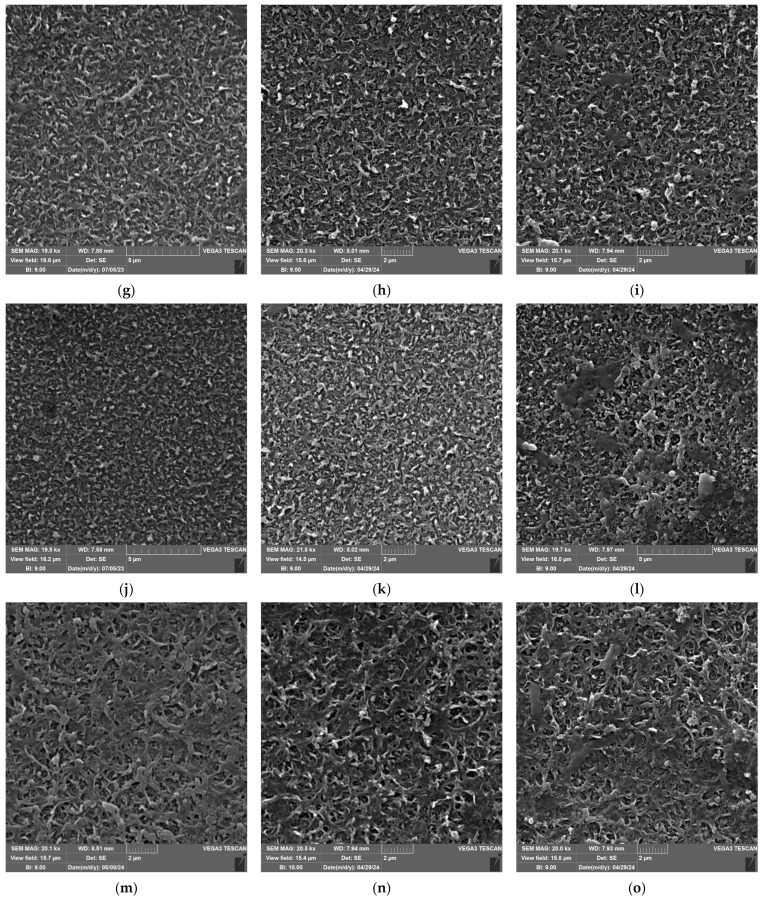
SEM micrographs of pristine (**a**) ACM1, (**d**) ACM5, (**g**) BW30LE, (**j**) UTC73AC, and (**m**) XLE; HEX-treated (**b**) ACM1, (**e**) ACM5, (**h**) BW30LE, (**k**) UTC73AC, and (**n**) XLE; and HDMA-treated (**c**) ACM1, (**f**) ACM5, (**i**) BW30LE, (**l**) UTC73AC, and (**o**) XLE membrane surfaces.

After plugging of the ACM1 membrane with both solutions, a noticeable change was observed in its nanostructure. The initial dense spherical nodular pattern shifted to a more irregular arrangement, resembling polymer strands with a leaf-like morphology (Figure 7a–c). Polymer strands with a leaf-like morphology of the pristine ACM5 membrane are distributed uniformly and densely across the upper surface. The micrographs following HDMA treatment show a consistent structure without any significant variation. Nevertheless, the ACM5 membrane after exposure to the HEX plugging solution shows a smoother surface with apparently less branched polymer strands compared to the pristine and the membrane after HDMA treatment (Figure 7d–f). For the BW30LE (Figure 7g,h) and UTC73AC (Figure 7j,k) membranes, there was no notable variance in structure evident in the micrographs post HEX treatment. Likewise, for the BW30LE membrane (Figure 7i), post-HDMA treatment did not result in any discernible alterations in surface morphology. However, after treatment with HDMA, the UTC73AC (Figure 7l) and XLE (Figure 7o) membranes exhibited visible alterations in morphology. It appears that HDMA plugging caused a shift in the leaf-like morphology structure of the PA membrane to a somewhat coagulated form in the UTC73AC and XLE membranes. The micrographs obtained subsequent to HEX treatment of the XLE membrane depict a higher incidence of branched strands in comparison to the pristine membrane.

### 3.6. Pore Size Distribution

**Figure 8:**
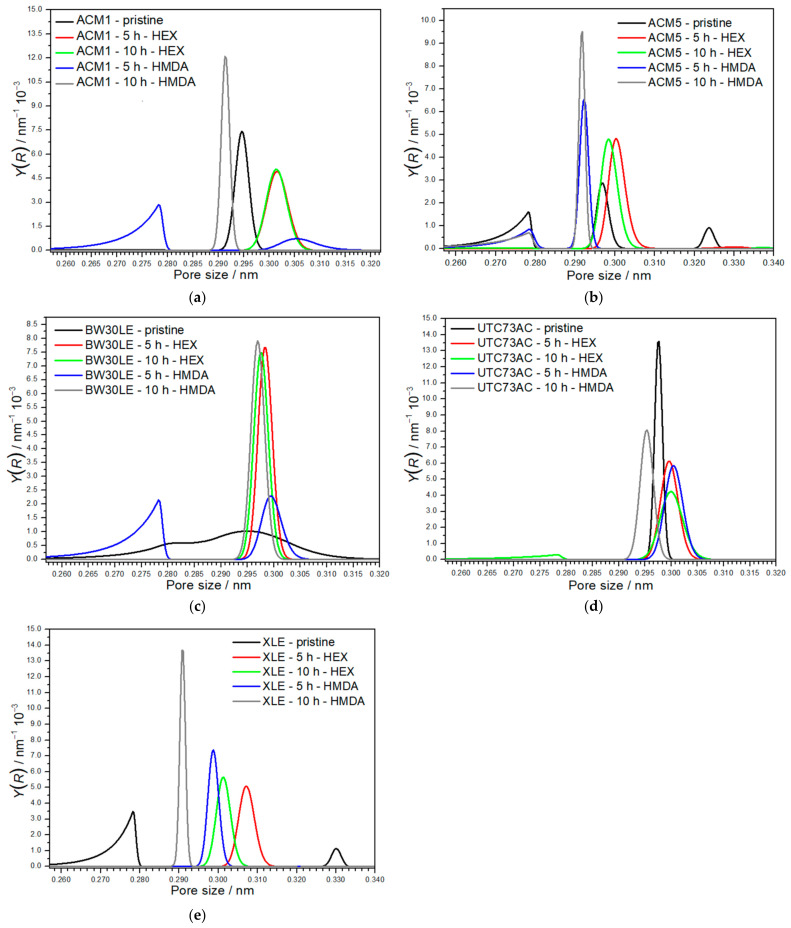
Pore size distribution of pristine and modified RO membranes: (**a**) ACM1, (**b**) ACM5, (**c**) BW30LE, (**d**) UTC73AC, and (**e**) XLE.

The porosity of ACM1, ACM5, BW30LE, UTC73AC and XLE membranes was analyzed before and after exposure to HEX (for 5 and 10 h) and HDMA (for 5 and 10 h), and the resulting pore size distributions (PSD) are shown in Figure 8. It is evident that the PSDs obtained vary for each membrane used. Initially, the pristine ACM1 membrane displays a unimodal distribution, with the peak of the curve situated within the range of 0.290–0.300 nm. However, exposure to HEX and HDMA induced changes in the pore size distribution. For HEX, the PSDs obtained after 5 and 10 h of exposure show similarities, albeit with reduced peak intensity and a slight shift of the curve maximum to the 0.286–0.309 nm range. Conversely, after a 5-h exposure of the ACM1 membrane to HDMA, a bimodal distribution is observed, with the first peak showing low intensity and broadness within the range of 0.259–0.281 nm and a broader peak occurring in the range of 0.297–0.315 nm. In addition, after 10 h of exposure to the HDMA solution, a different pore distribution was obtained compared to 5-h exposure and the pristine membrane. A narrow, sharp curve with higher intensity is observed in the range of 0.289–0.294 nm (Figure 8a). The pristine ACM5 membrane exhibits a trimodal distribution characterized by a broader and less intense first peak within the 0.258–0.281 nm range compared to the second peak falling in the 0.291–0.303 nm range, and a smaller third peak situated within the 0.319–0.328 nm range. Similar to the ACM1 membrane, comparable peaks with the same intensity in the range of 0.291–0.309 nm were obtained after 5 and 10 h of exposure to the HEX solution. However, a bimodal distribution with two peaks emerged after 5-h exposure to HDMA. The initial peak with reduced intensity is in the range of 0.258–0.282 nm, while the subsequent peak with increased intensity, lies within the range of 0.286–0.297 nm. Nevertheless, following a 10-h exposure to the HDMA solution, a higher intensity peak emerged within the range of 0.287–0.295 nm region (Figure 8b). In contrast to the other membranes, the PSD curve obtained of the pristine BW30LE membrane deviates and shows a significantly broader distribution, ranging from 0.259 to 0.318 nm. Following exposure to HEX (for 5 and 10 h) and HDMA solution (for 10 h), narrow peaks with high intensity within the range of 0.291–0.304 nm were observed. However, in the case of the 5-h exposure to the HDMA solution, the resulting PSD exhibited a bimodal distribution with two peaks of medium intensity in the range of 0.258–0.281 nm and 0.292–0.308 nm (Figure 8c). The analysis of the pore size distribution performed on the pristine UTC73AC membrane shows a unimodal distribution with a prominent peak observed within the 0.295–0.300 nm range. However, with the exception of the 10-h exposure to HEX, all treatments resulted in a unimodal distribution of pore sizes. In particular, following a 5-h exposure to HEX, a peak of approximately three times lower intensity compared to the pristine membrane occurred in the 0.294–0.306 nm range. After a 10-h exposure, a bimodal pore size distribution was observed, characterized by a smaller peak with significantly lower intensity in the 0.256–0.280 nm range, while a peak with three times lower intensity was found in the 0.295–0.305 nm range compared to the pristine membrane. After a 5–hour treatment with HDMA, the pore size distribution was similar to that observed with HEX treatment of the same duration and the emerged peak can be seen between 0.294 and 0.306 nm. However, following a 10-h treatment with HDMA, a distinct peak appeared in the range of 0.291–0.300 nm (Figure 8d). The PSD obtained for the pristine XLE membrane shows a bimodal distribution. The initial peak, which is in the range of 0.258–0.281 nm, displays a broader distribution and a slightly higher intensity compared to the second peak, which is situated within the range of 0.327–0.335 nm. Each of the mentioned treatments had a distinct impact on the membrane; however, in all instances, a unimodal distribution of pore sizes was formed after treatment. With the HEX solution (5 h), a peak appeared in the range of 0.300–0.315 nm, while with HEX (10 h) a peak appeared within the range of 0.295–0.308 nm. After treatment with HDMA (5 h), a peak in the range of 0.294–0.304 nm was observed, and with HDMA (10 h), a high intensity peak appeared in the range of 0.287–0.295 nm (Figure 8e). The pore size distribution indirectly determined by this methodology agrees with the PSD by other methods [29]. It is also important to note that although it is evident that plugging affects PSD, the change in pore size remains within a very narrow range.

## 4. Discussion

**Figure 9:**
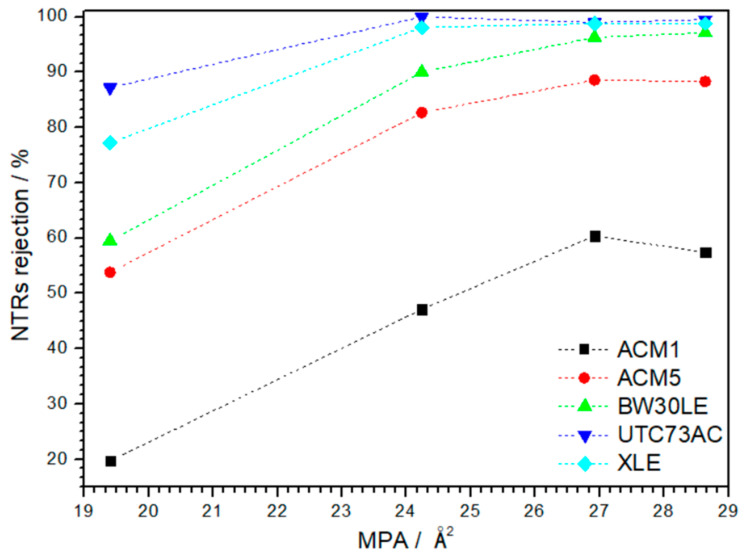
Rejection of NTRs by the selected pristine RO membrane as a function of MPA.

The main objective of this investigation was to improve the rejection efficiency of five TFC-PA membranes, each characterized by different morphological parameters and the physical and chemical properties of the polymer, specifically targeting NTRs. The separation capability was tested with NaCl and four NTRs with different MPAs. All selected pristine membranes showed high NaCl rejection, i.e., the rejection of ACM1, ACM5, BW30LE, UTC73AC and XLE membranes was 99.1, 98.7, 99.0, 98.5 and 98.7%, respectively (Figure 3). Moreover, each of the selected membranes showed a different rejection towards NTRs (Figure 4), whereas rejection was clearly influenced by geometrical factors such as thickness, area and porosity, as well as the physical and chemical characteristics of the polymer. These factors include polarity, fixed charge content, degree of cross-linking and water content (swelling) [14]. The fluctuations in the rejection of NTRs are particularly noticeable with the smallest NDMA molecule. Additionally, the rejection of the four NTRs increased with the increase in the MPA in all selected RO membranes, emphasizing the influence of the size exclusion mechanism in the removal of NTRs (Figure 9). Since NTRs are small, uncharged molecules at pH 6–8, steric hindrance is an important factor in their rejection [22].

Furthermore, it was found that the rejection of NDMA, which is characterized by the smallest MPA among the NTRs, was optimal for the pristine UTC73AC membrane, reaching 87.2% rejection. In contrast, the pristine ACM1 membrane exhibited a significantly lower removal efficiency, with a rejection rate of only 19.7%. Moreover, the ACM5, BW30LE and XLE membranes showed NDMA rejection efficiencies of 53.8%, 59.5% and 77.2%, respectively. The surface morphology and overall porosity of a membrane can affect the rejection of NDMA. Membranes with higher porosity may have more interconnected pathways that could facilitate the passage of smaller molecules. Variations in pore size distribution among different pristine membranes may be responsible for their different ability to reject NDMA. Typically, membranes display two p*K*_a_ values near pH 5–6 and 8–9, which are associated with different pore types and their respective local environments in the polyamide. The higher p*K*_a_ value, around 8–9, corresponds to smaller “network” pores below 0.25 nm in the dense polyamide. The lower p*K*_a_ value, near 5–6, is associated with larger “aggregate” pores above 0.35 nm located between aggregated clusters of the denser polymer [29]. Membranes with a narrow pore size distribution and smaller average pore sizes provide a more uniform barrier against small molecules. This uniformity may ensure that fewer NDMA molecules can pass through the pores, resulting in higher rejection rates. In this study, two membranes showed a unimodal distribution of pores, ACM1 with the peak of the curve in the range of 0.290–0.300 nm and UTC73AC with a prominent peak in the range of 0.295–0.300 nm. Compared to the others, the BW30LE membrane showed a significantly broader unimodal distribution in the range of 0.259 to 0.318 nm. The ACM5 membrane exhibited a trimodal distribution characterized by a broader and less intense first peak in the range of 0.258–0.281 nm, a second peak in the range of 0.291–0.303 nm and a smaller third peak in the range of 0.319–0.328 nm. The XLE membrane showed a bimodal distribution with a first peak in the range of 0.258–0.281 nm and a second peak in the range of 0.327–0.335 nm. However, when comparing the obtained NDMA rejections with the corresponding pore size distribution curves, significant differences become apparent in some cases. Specifically, while the ACM1 and the UTC73AC membranes show a similar pore size distribution (Figure 8a,d), there is a notable discrepancy in NDMA removal, i.e., the rejection differed by approximately 77%. This observation could indicate that several mechanisms are responsible for the removal of small neutral molecules such as NDMA. The interaction between solutes and a porous membrane is often cited as an important mechanism for rejection, i.e., certain interactions, such as hydrogen bonding and electrostatic interactions between specific functional groups of the solute and membranes, play a crucial role [30,31,32]. Polyamide membranes generally feature chemical functional groups like –CONH_2_, –COOH and –NH_2_ on their surface [14,33,34]. The latter two groups indicate the presence of unreacted carboxylic and amine groups, which act as fixed charges. In addition, studies have shown that polyamide contains around 0.3–0.6 M of carboxylic groups, which corresponds to a cross-linking of 94–96%, with 8–15% of the trimesoyl chloride (TMC) units remaining uncross-linked [14,35,36]. In the study conducted by Widjaya et al., it was shown that the polyamide structure of the BW30LE membrane had an oxygen/nitrogen (O/N) ratio of 1.1, which corresponds to a cross-linking degree of approximately 87% [37]. This indicates the presence of residual –COOH and –NH_2_ groups from unreacted monomers. In contrast, a study by Tang et al. reported that the O/N ratio for the XLE membrane was 1.0, suggesting a fully cross-linked structure [38]. It is important to note that the structural differences, including the different proportions of functional groups within the PA layer, can significantly affect the interactions between solutes and the PA layer, thus affecting solute permeation and the rejection factor. However, the role of certain chemical functional groups on membrane surfaces remains largely undisclosed because of the physical and chemical heterogeneity arising from the interfacial polymerization process that forms the thin active (selective) barrier in RO membranes. Apart from the physical and chemical properties of the polymer, rejection of membranes depends on the size and geometry of the molecules of the solute as well as the nature of the solute, i.e., the charge and polarity. In a separate study using compounds with different functional groups, Kiso [39] demonstrated that polar groups within compounds have different effects on adsorption. In particular, it was observed that the influence of polar groups on adsorption between cellulose acetate and the solute molecule decreases in the following order: –C(O)O– > –CO > HCON > CH_3_CON > –OH– > –O–. To further investigate the aforementioned, rejection of the non-ionizable compound 1,3-dioxolane, which is characterized with a comparable MPA of 19.2 Å^2^ to that of NDMA, was investigated.

**Figure 10:**
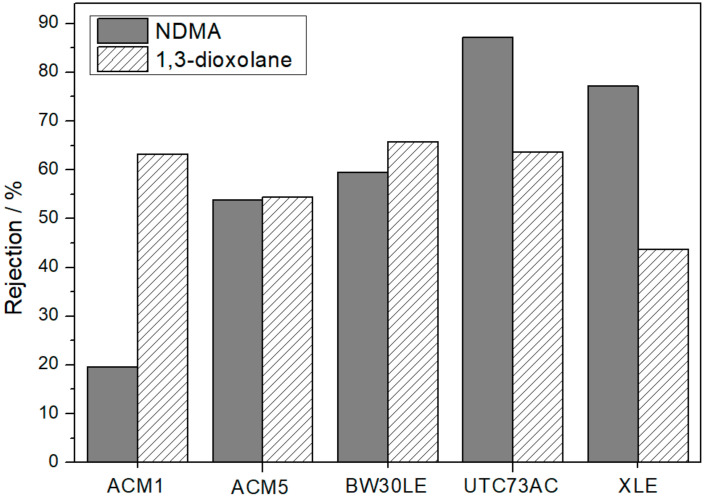
The rejection of NDMA and 1,3-dioxolane for selected pristine RO membranes.

The graphical representation (Figure 10) shows that there is no significant difference in the removal of 1,3-dioxolane and NDMA in the case of ACM5. In addition, it can be seen that the ACM1 and BW30LE membranes show an increased rejection of 1,3-dioxolane. Conversely, the UTC73AC and XLE membranes demonstrate a significantly lower rejection of 1,3-dioxolane compared to NDMA.

The lower rejection of NDMA observed in ACM1 and BW30LE membranes may be attributed to the greater tendency of NDMA to adhere to the membrane material via hydrogen bonding [22]. This allows NDMA to permeate the membrane, consequently yielding negative rejection results. Comparable to the permeation of water through a reverse osmosis membrane, which is facilitated by hydrogen bonds with the polymer, solutes that have a greater propensity to hydrogen bond could displace the water molecules to some degree, thereby reducing the flux. In the study by Zhang et al., it was shown that the H-bonds between water and –COOH have the longest lifetime, suggesting that the –COOH group of the PA membrane exerts the strongest attraction to the surrounding water molecules compared to (NH)CO − water and NH_2_ − water H-bonds [40]. Depending on the membrane, however, the membrane morphology can differ, i.e., the degree of cross-linking can vary greatly, which leads to different proportions of the individual functional groups. Within fully cross-linked polyamide skin layer, the hydrogen atom on the amide (CO–NH–) or free amine (NH_2_–) functional groups can serve as a hydrogen bond donor. While the keto group (=O) acts as a hydrogen bond acceptor with a partial negative charge (δ–), the nitrogen atoms in nitrogen-containing molecules also act as hydrogen bond acceptors, thereby increasing the potential for hydrogen bonding [41]. In order to theoretically determine the relevance of the thesis, we have calculated the strength of the hydrogen bond between the molecules of NDMA, 1,3-dioxolane and the monomeric unit of polyamide. The calculated value of hydrogen bonding between –CONH_2_ within the PA structure and NDMA was 32.6 kJ mol^−1^, whereas it was 26.7 kJ mol^−1^ between polyamide and 1,3-dioxolane. Consequently, NDMA shows a higher affinity to polyamide through hydrogen bonding, which is approximately 18% stronger than that of 1,3-dioxolane. The obtained value of hydrogen bond strength between NDMA and PA and 1,3-dioxolane and PA is consistent with the results for ACM1 and BW30LE membranes and supports the given premise. However, in the case of the ACM5 membrane, the deviation in the rejection rates of NDMA and 1,3-dioxolane was not observed and was not considered significant. In contrast, an opposite trend was observed for the UTC73AC and XLE membranes. In particular, the rejection of NDMA was significantly higher compared to 1,3-dioxolane. This phenomenon is likely attributable to the different physicochemical characteristics of the membranes. Specifically, the quantity and presence of various functional groups resulting from differences in cross-linking, polarity and water content may contribute to this reverse effect. Consequently, UTC73AC and XLE showed higher rejection of NDMA compared to 1,3-dioxolane.

**Figure 11:**
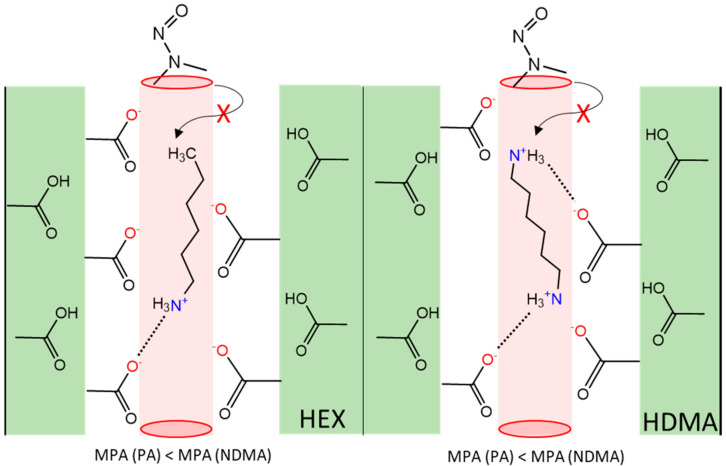
Schematic representation of molecular plugging in PA-RO membranes.

Considering that most of the pristine membranes showed lower NDMA rejection, the main objective of this study was to improve the rejection by incorporating additional moieties, i.e., molecular plugs, into the selective PA layer of the membranes. A schematic representation of molecular plugging in PA-RO membranes is shown in Figure 11.

Plugging the ACM1, ACM5 and BW30LE membranes with HEX and HDMA led to a decrease in water permeability with a simultaneous increase in NTR rejection.

**Figure 12:**
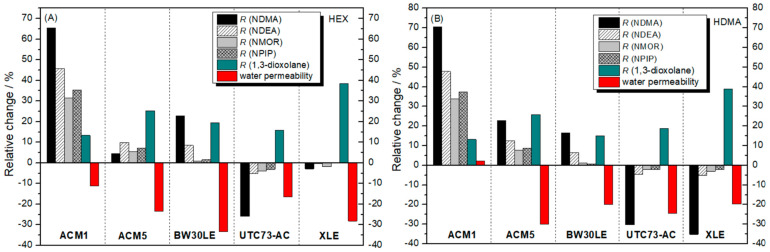
Relative change in water permeability and rejection of NDMA and 1,3-dioxolane after treatment with (**A**) HEX and (**B**) HDMA.

After a 10-h of plugging with HEX, the rejection factors of ACM1, ACM5 and BW30LE membranes increased for NDMA (65.5%, 4.6% and 22.9%, respectively), NDEA (45.7%, 9.9% and 8.6%, respectively), NMOR (31.5%, 5.5% and 0.8%, respectively) and NPIP (35.4%, 7.2% and 1.5%, respectively) compared to the pristine membranes (Figure 12A and Table 4). At the same time, the water permeability of these membranes decreased by 11.1%, 23.5% and 33.2%, respectively (Figure 12A). The PSD results are consistent with the rejection factors determined for each compound (Table 4). For the ACM1 membrane in particular, the PSD curves show that after exposure to the HEX solution for 10 h, pores with a size of 0.301 nm predominate. These pores are not significantly larger than the initial ones, and the intensity of the resulting peak is 32% lower than that of the pristine membrane, suggesting a possible reduction in the effective pore number. In addition, for the ACM5 membrane, after plugging, the initial trimodal distribution of pores became a unimodal distribution. It is evident that the pores with a maximum at 0.324 nm are no longer present and a new peak with a maximum at 0.298 nm appeared. In the case of the BW30LE membrane, the PSD shows a unimodal curve with a maximum at 0.298 nm instead of the previously very broad pore distribution in the range of 0.258–0.317, indicating a much narrower distribution of pores after plugging. However, plugging of the UTC73AC and XLE membranes resulted in reduced rejection of NTRs. The rejection of NTRs decreased for NDMA (25.9% and 2.9%, respectively) (Figure 12A), NDEA (5.1% and 0.3%, respectively), NMOR (4% and 1.7%, respectively) and NPIP (3.1% for UTC73AC) despite the resulting visible distribution of smaller pores (Table 4). Similarly, the water permeability of the UTC73AC and XLE membranes also decreased by 16.3% and 28.1%, respectively (Figure 12A), supporting the above assumption of a reduction in pore size. For comparison, the rejection of 1,3-dioxolane was investigated. For the ACM1, ACM5 and BW30LE membranes, the rejection of 1,3-dioxolane improved by 13.4%, 25.2% and 19.6%, respectively (Figure 12A), after plugging, which is consistent with the increased rejection of NDMA. Moreover, while the rejection of NDMA decreased specifically for the UTC73AC and XLE membranes, the rejection of 1,3-dioxolane increased by 15.7% and 38.4%, respectively, after plugging, which is largely inconsistent with the results for NDMA (Figure 12A).

After subjecting the ACM1, ACM5 and BW30LE membranes to HDMA for 10 h, the rejection rates for NDMA increased significantly: 70.6%, 22.8% and 16.6%, respectively (Figure 12B). Similarly, rejection of NDEA (47.9%, 12.6% and 6.5%, respectively), NMOR (33.9%, 7.8% and 1.1%, respectively) and NPIP (37.5%, 8.7% and 0.7%, respectively) also increased significantly for all three membranes (Table 4). At the same time, the water permeability decreased by 29.9% for ACM5 and by 19.8% for BW30LE (Figure 12B). Surprisingly, the water permeability of ACM1 increased by 2.3% compared to the pristine membrane. Furthermore, the rejection of 1,3-dioxolane for the ACM1, ACM5 and BW30LE membranes improved by 13.4%, 25.9% and 15%, respectively, following HDMA plugging, which is consistent with the increased rejection of NDMA and suggests that smaller pores predominate within the PA layer after treatment with HDMA (Figure 12B). In agreement with previous observations, plugging of the UTC73AC and XLE membranes resulted in lower rejection of NTRs, despite the presence of smaller pores. In particular, rejection significantly decreased for the smallest NDMA (30.3% and 35.2%, respectively) (Figure 12B), and less for larger molecules NDEA (4.6% and 5.1%, respectively), NMOR (2.2% and 3.0%, respectively) and NPIP (2.2% and 2.1%, respectively) (Table 4). In addition, the water permeability of the UTC73AC and XLE membranes decreased by 24.5% and 19.7% respectively. However, the rejection of 1,3-dioxolane increased by 18.9% and 38.9%, respectively for UTC73AC and XLE membranes after plugging with HDMA (Figure 12B). This inconsistency in conjunction with the observations following plugging with HEX suggests that altered physicochemical parameters may affect rejection ability of the membranes.

Overall, findings indicate that the use of HEX and HDMA as molecular plugs effectively enhances the rejection of NTRs and 1,3-dioxolane for ACM1, ACM5 and BW30LE membranes, albeit at the expense of water permeability. There is a notable inverse relationship between water permeability and the rejection obtained, which is expected if the hole size of the free volume is reduced (Figure 12A,B). HEX and HDMA contain ${–NH}_{3}^{+}$ groups that can interact electrostatically with the functional groups present, thereby narrowing the pore size in the PA layer and restricting the permeation of water, 1,3-dioxolane and NTRs. Additionally, a slightly higher trend in the removal of 1,3-dioxolane and NTRs is observed when HDMA is used as a molecular plug. This phenomenon may be attributed to the presence of two ${–NH}_{3}^{+}$ groups in HDMA, which double the probability of attaching plugging molecules to the “pore walls” and possibly increase the probability of immobilization within the pores.

In the case of UTC and XLE membranes, plugging with HEX and HDMA changed the PSD due to the binding of the molecular plugs, which increased the separation of 1,3-dioxalane. However, these molecular plugs did not improve the rejection of NDMA. This might be due to the different membrane morphologies; the XLE membrane is more cross-linked than the BW30LE. In particular, the XLE membrane is denser and has fewer free carboxylic acid groups. Since the carboxylic acid group exerts a strong electrostatic attraction to the surrounding molecular plugs carrying ${–NH}_{3}^{+}$, its absence may reduce the likelihood of the molecular plugs being bound by electrostatic interactions and therefore, the positive effect will not manifest itself as in the case of BW30LE. Furthermore, with the introduction of the molecular plugs along with reduced access to the functional groups responsible for binding the molecular plugs via electrostatic interactions, additional groups ${–NH}_{3}^{+}$/${–NH}_{2}$ could be incorporated into the membrane, potentially increasing the likelihood of hydrogen bonding of NDMA with the added groups and thus allowing easier permeation within the interconnected pores in the PA layer. This trend is also reflected in the fact that the rejection of NDMA is lower after the incorporation of HDMA with two ${–NH}_{3}^{+}$ groups compared to HEX. Therefore, it can be concluded that the binding of molecular plugs in XLE and UTC73AC significantly differs from other membranes, leading to the conclusion that changes in the PA layer may affect NTR permeation within the RO membrane.

Based on the summarized results, including rejection rates, water permeability, and pore size distribution (PSD) curves, it is evident that both molecular plugs, HEX and HDMA, were effectively integrated into the PA layer.

**Figure 13:**
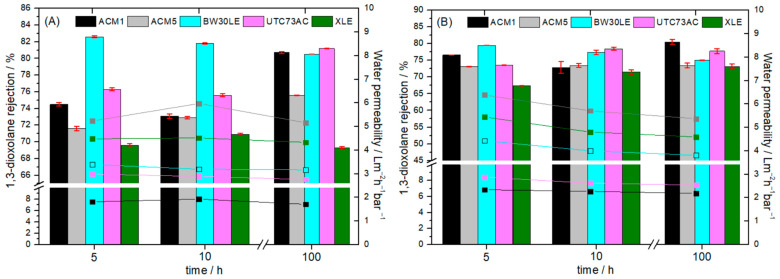
The rejection of 1,3-dioxolane and the pure water permeability of the (**A**) HEX- and (**B**) HDMA-plugged RO membranes over a 100-h period. The error bars indicate the mean values and ranges of the duplicated separation results.

In addition, the long-term separation performance of the RO membranes was evaluated with 1,3-dioxolane over a period of 100 h, during which water permeability and the rejection of 1,3-dioxolane were measured. At a constant transmembrane pressure of 15 bar, water permeability remained stable and rejection factors were consistently high, especially when compared to pristine membranes (Figure 13). This finding could indicate that the molecular plugs were immobilized, and the 100-h test could imply that the molecular plugs do not easily detach from the RO membrane during treatment.

However, while the IR spectra clearly show bands associated with aromatic polyamides, it is uncertain whether the bands at 3309 cm^−1^ and 1613 cm^−1^ and 797 cm^−1^, characteristic of primary amines are present due to a possible overlap with membrane-related bands. Additionally, a notable decrease in the contact angle can be seen in Figure 5. This reduction can be explained by the sorption of HEX and HDMA, whereas amines that are not electrostatically bound within the PA layer possibly align themselves so that their hydrophobic tails adhere to the membrane and the hydrophilic amine groups point outwards, causing a reduction in the contact angle values [23]. A different orientation of the amine molecules can lead to the opposite result and cause a higher contact angle, as observed with the UTC73AC membrane. In addition, the change in contact angle might be attributed to changes in the morphology of the membrane. Typically, a rougher surface of the RO membrane leads to a smaller contact angle. This occurs because a rough surface increases the solid–liquid interfacial free energy, and a higher interfacial free energy favors a decrease in the contact angle [42]. SEM micrographs show that in certain cases, such as ACM1, exposure to the plugging solution results in a more irregular arrangement resembling polymer strands. These slight morphological changes, possibly associated with increased roughness, may also influence contact angle measurements.

## 5. Conclusions

The initial phase of this study involved assessing the rejection of NTRs by different RO membranes. It was found that the efficiency of rejection strongly depends on the type of RO membrane used. Regardless of the membrane type, it was observed that the size exclusion mechanism plays an important role in the separation of NTRs with different MPA. However, the lower initial separation efficiency is largely attributed to the interaction between NTRs and the PA layer through the formation of hydrogen bonds, which is particularly noticeable for NDMA. The contribution of these mechanisms to the rejection of NTRs appears to be influenced by the structure of the PA layer, such as the PSD and the cross-linking.

In the second part of the study, the membranes were modified with molecular plugs and their impact on membrane morphology (PSD) and membrane performance (permeability, rejection) was investigated. The treatment with molecular plugs altered the PSD and effective pore number, resulting in a reduction in the membrane water permeability (5–33%). Despite the decline, water permeability remained acceptable for industrial purposes. HEX and HDMA had a positive effect on NTRs rejection with the ACM1, ACM5 and BW30LE membranes. In particular, the rejection of NDMA, the smallest NTRs molecule, by ACM1 membrane was improved by 65.5% and 70.6% after treatment with HEX and HDMA, respectively. However, for the UTC73AC and XLE membranes, NTRs removal decreased after treatment with molecular plugs. The different effects of molecular plugs on NTR separation could be due to differences in membrane structure and the availability of –COOH groups, which facilitate the binding of HEX and HDMA through electrostatic interactions with ${–NH}_{3}^{+}$ groups. Furthermore, the size exclusion mechanism remained significant for the separation of NTRs with different MPAs. Finally, it was discovered that the less toxic HDMA molecular plug exhibited greater rejection toward NTRs.

## Figures and Tables

**Table 1:** The physico-chemical properties of the selected compounds.

Compound	Chemical Structure	Molecular Formula	Molecular Weight/g mol^−1^	Minimal Projection Area (MPA)/Å^2^	p*K*_a_	log *K*_ow_
NDMA	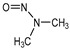	C_2_H_6_N_2_O	74.08	19.4	3.52	−0.57
NDEA	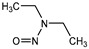	C_4_H_10_N_2_O	102.14	24.2	3.32	0.34
NMOR	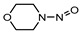	C_4_H_8_N_2_O_2_	116.12	26.9	3.14	−0.44
NPIP	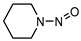	C_5_H_10_N_2_O	114.08	27.2	3.30	0.72
1,3-dioxolane	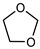	C_3_H_6_O_2_	74.08	19.2	N.I.	−0.37

**Table 2:** Properties of commercially available RO membranes according to the manufacturer’s specifications.

	DOW-FILMTEC™BW30LE	Toray™ UTC-73AC	TriSep™ ACM1	TriSep™ ACM5	DOW FILMTEC™ XLE
Feed	Brackish Water	Brackish Water	Brackish Water	Brackish Water	Brackish Water
Type	Low Energy	High Rejection, Low Energy, Cl Resistant	“Tight”	Low Pressure, High permeability	Extra-Low Energy
pH Range (25 °C)	2–11	2–11	2–11	2–11	2–11
Rejection (NaCl)/%	99.0%	99.8%	99.5%	98.5%	98.7%
Pore size/MWCO	N/A	N/A	N/A	N/A	N/A
Polymer	Polyamide-TFC	Polyamide-TFC	Polyamide-TFC	Polyamide-TFC	Polyamide-TFC
Water permeability/L m^−2^ h^−1^ bar^−1^	4.06–5.05	3.37	2.74	6.7	6.5–8.1

**Table 3:** The physico-chemical properties of the selected amines.

Compound	Chemical Structure	Molecular Formula	Molecular Weight/g mol^−1^	Min Projection Area (MPA)/Å^2^	p*K*_a_	Log *D* at pH 7.4	Solubility/g L^−1^
HMDA	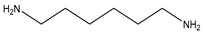	C_6_H_16_N_2_	116.208	21.03	10.51	−5.16	100
HEX	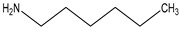	C_6_H_15_N	101.2	20.7	10.5	−1.02	12

**Table 4:** The rejection of NTRs and 1,3-dioxolane of selected pristine and modified RO membranes, with the calculated MPA of the pores.

Membranes	MPA/Å^2^				Rejection/%				
					DIOX	NDMA	NDEA	NMOR	NPIP
					19.20	19. 40	24.24	26.92	28.64
Pristine									
ACM1	27.3				63.3	19.7	47.1	60.4	57.4
ACM5	24.3	27.7		33.0	54.5	53.8	82.7	88.6	88.3
BW30LE	21.2–31.8				65.8	59.5	90.0	96.3	97.1
UTC73AC		27.9			63.7	87.2	100.0	99.0	99.5
XLE	24.3		34.2		43.7	77.2	98.1	98.8	98.8
HEX									
ACM1	28.4				73.1	57.0	86.9	88.2	89.0
ACM5	27.9				72.9	56.4	91.8	93.7	95.2
BW30LE	27.9				81.8	77.2	98.5	97.1	98.5
UTC73AC	24.3		28.1		75.6	69.3	95.1	95.2	96.5
XLE	28.4				70.9	75.0	97.8	97.1	99.0
HDMA									
ACM1	26.6				72.9	66.8	90.4	91.4	91.9
ACM5	24.3		26.8		73.5	69.7	94.7	96.1	96.7
BW30LE	27.7				77.4	71.3	96.3	97.4	97.7
UTC73AC	27.3				78.5	67.0	95.6	96.8	97.4
XLE	26.6				71.5	57.1	93.4	95.9	96.8

## Data Availability

The original contributions presented in the study are included in the articles, further inquiries can be directed to the corresponding author.
